# 1498. Geographic differences in weight change on dolutegravir: a prospective cohort study

**DOI:** 10.1093/ofid/ofad500.1333

**Published:** 2023-11-27

**Authors:** Geoffrey Chen, Richard Migisha, Winnie R Muyindike, Taing N Aung, Victoria Nanfuka, Nimusiima Komukama, Nomathemba Chandiwana, Gugulethu Shazi, Mahomed-Yunus S Moosa, Ravindra K Gupta, Deenan Pillay, Vincent Marconi, Bethany Hedt-Gauthier, W D Francois Venter, Mark J Siedner, Suzanne M McCluskey, Jennifer Manne-Goehler

**Affiliations:** Massachusetts General Hospital, Boston, Massachusetts; Mbarara University of Science and Technology, Mbarara, Mbarara, Uganda; Mbarara University of Science and Technology, Mbarara, Mbarara, Uganda; Massachusetts General Hospital, Boston, Massachusetts; Mbarara University of Science and Technology, Mbarara, Mbarara, Uganda; Mbarara University of Science and Technology, Mbarara, Mbarara, Uganda; University of the Witwatersrand, Johannesburg, Gauteng, South Africa; Africa Health Research Institute, Durban, KwaZulu-Natal, South Africa; University of KwaZulu-Natal, Durban, KwaZulu-Natal, South Africa; University of Cambridge, Cambridge, England, United Kingdom; Africa Health Research Institute, Durban, KwaZulu-Natal, South Africa; Emory University, Atlanta, Georgia; Harvard Medical School, Boston, Massachusetts; University of the Witwatersrand, Johannesburg, Gauteng, South Africa; Africa Health Research Institute, Durban, KwaZulu-Natal, South Africa; Massachusetts General Hospital, Boston, Massachusetts; Massachusetts General Hospital, Boston, Massachusetts

## Abstract

**Background:**

People with HIV (PWH) on integrase inhibitors may be at increased risk of excess weight gain, but it is unclear if this risk is consistent across settings. Our study objective was to compare weight change over 48 weeks among PWH in Uganda and South Africa.

**Figure 1.**
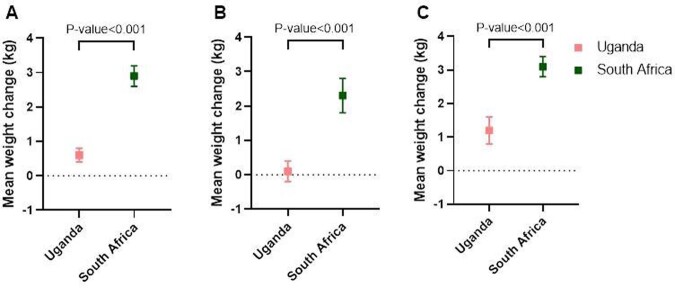
Mean weight change (kg) over 48 weeks among DISCO participants overall (A), among men (B), and among women (C).

**Methods:**

The Population Effectiveness of Dolutegravir Implementation in Sub-Saharan Africa (DISCO) study is a prospective observational cohort of PWH in routine clinical care at public-sector HIV clinics in Uganda and South Africa. Inclusion criteria were as follows: PWH >18 years old, on NNRTI-based first-line ART for >6 months, and switched to tenofovir disoproxil fumarate, lamivudine, and dolutegravir) by clinic staff. We measured the primary outcomes of weight (in kilograms [kg]) and waist circumference (WC, in centimeters [cm]) at enrollment, 24 weeks, and 48 weeks after switch. The primary outcomes were (1) weight change (kg) and (2) change in WC (cm). We used a linear mixed-effect regression model, adjusted for age, sex, education, duration on ART, and the interaction of study site and visit, to estimate weight.

**Results:**

428 individuals in Uganda and 387 in South Africa had data available. The mean weight change over 48 weeks was 0.6 kg [95% CI: 0.1-1.0] in Uganda compared to 2.9 kg [2.4-3.4] in South Africa (p< 0.001); men had significantly smaller mean weight changes than women did in both countries (Figure 1). After adjustment, PWH in South Africa gained significantly more weight than those in Uganda. In participants with available waist data (277 in Uganda and 402 in South Africa), the mean change in WC was significantly greater among those in South Africa (2.3 cm [1.4-3.2]) than those in Uganda (0.8 cm [0.0-1.5]) (p< 0.017).

**Conclusion:**

PWH in South Africa experienced greater weight gain than in Uganda, suggesting substantial heterogeneity in this risk across settings. Strategies to address obesity risk in PWH should account for regionality.

**Disclosures:**

**W D Francois Venter, MD, FCP, PhD**, Gilead Sciences: Grant/Research Support|South African Medical Research Council: Grant/Research Support|Unitaid: Grant/Research Support|USAID: Grant/Research Support|ViiV Healthcare: Grant/Research Support **Mark J Siedner, MD, MPH**, Viiv Healthcare: Grant/Research Support

